# The gender gap in academic surgery: the electronic health record

**DOI:** 10.1186/s12893-025-02808-w

**Published:** 2025-02-28

**Authors:** Sarah Y. Bessen, Sean Tackett, Carolyn M. Jenks, C. Matthew Stewart, Maria Oliva-Hemker, Jennifer K. Lee

**Affiliations:** 1 Department of Otolaryngology, Head & Neck Surgery, Johns Hopkins University School of Medicine (JHUSOM), Baltimore, USA; 2 Department of Medicine, JHUSOM, Baltimore, USA; 3 Biostatistics, Epidemiology, and Data Management Core, JHUSOM, Baltimore, USA; 4 Department of Pediatrics, JHUSOM, Baltimore, MD USA; 5 Office of Faculty, JHUSOM, 1800 Orleans Street, Suite 6321, Baltimore, MD 21287 USA; 6 Department of Anesthesiology and Critical Care Medicine, JHUSOM, Baltimore, USA

**Keywords:** Gender, Equity, Electronic health record, Burnout, Surgery

## Abstract

**Background:**

The electronic health record (EHR) contributes to burnout, and excessive EHR work impedes productivity. The scope of potential gender discrepancies in EHR burden among surgeons is unclear. Because clinical operations and workflow vary by institution, EHR research must be conducted at multiple centers. Identifying modifiable factors that influence how surgeons use the EHR would support strategies that mitigate gender discrepancies. We hypothesized that gender differences in EHR use would be related to EHR support from clinical team members, patient message volume, and documentation length.

**Methods:**

We retrospectively evaluated EHR use by surgeons from multiple specialties in eight departments at a large academic center from January to December 2023. Data about clinical and EHR workload were collected using a provider efficiency tracking tool. These data included the amount of time surgeons spent working in the EHR outside of regular work hours overall and on individual tasks such as responding to messages, clinical review, and orders; the number of messages received and their source; proficiency with documenting in the EHR; and receipt of assistance with EHR documentation and orders from clinical team members.

**Results:**

EHR use was analyzed from 323 surgeons (32% women). After adjusting for specialty, women spent more time than men working in the EHR outside of scheduled clinical hours (mean difference: 7.8 min/day; *p =* 0.001) and 7 AM to 7 PM (3.8 min/day; *p =* 0.007) despite no difference in clinical workload. Women spent more time on notes (1.8 min/appointment; *p <* 0.001), messages (0.7 min/appointment; *p =* 0.004), and clinical review (0.6 min/appointment; *p =* 0.032) than men. However, there were no surgeon gender differences in clinical support for EHR work, number of patient messages, note length, or proficiency with the EHR.

**Conclusion:**

Women surgeons had a greater EHR burden than men at our institution. Assuming 16 clinical days/month, 8 min/day of additional EHR work outside of scheduled clinical hours for women translates to approximately 26 h/year of lost time relative to men. Unequal EHR work may increase the gender gap in career advancement and exacerbate burnout for women. Future research should include prospective multi-center trials to clarify the extent of the surgical EHR gender gap.

## Background

Demands for clinical productivity are increasing in hospitals and surgical centers nationwide. Requirements for additional documentation in the electronic health record (EHR) compound the risk of physician burnout [1]. Studies that evaluate surgical and nonsurgical physicians together suggest that women have higher EHR workloads than men and that these gender-based EHR differences may be increasing over time [2–6]. 

Separately evaluating the EHR workload for surgeons is essential because differences between the surgical and medical fields in their daily workflow and nature of patient interactions could drive unique patterns of EHR use. Physician gender and EHR research has largely focused on primary care and other medical specialties. To our knowledge, there have been only two studies about surgeons, and it is unknown to what extent the findings are generalizable [7, 8]. Institutional differences in expectations for clinical productivity, case complexity, support from clinical team members, and work culture and environment each influence how and when surgeons use the EHR. If studies collectively show that women surgeons have an unequal distribution of EHR work relative to men at many institutions across the United States, then concerted effort at a health system level may be needed to mitigate the problem.

Gender differences in work can have long-term consequences. For example, women with a high EHR burden may have little time for professional and leadership development and activities that lead to promotion or salary raises. Such barriers could contribute to inequity in leadership, such as the low proportion of women surgeons in department chair positions [9] and persistent gender discrepancies in salary [10]. Modifiable and nonmodifiable factors associated with EHR work must also be identified to develop strategies that reduce the EHR burden.

In this study, we compared how women and men surgeons conduct their EHR documentation at a large academic medical center. We hypothesized that gender differences in EHR use would be related to differences in support for EHR work from clinical team members, patient message volume, and note length.

## Methods

### Study population

We conducted a retrospective, cross-sectional study of all full-time faculty surgeons from 8 surgical departments (otolaryngology, general surgery, neurosurgery, gynecologic surgery, ophthalmology, orthopedic surgery, plastic surgery, and urology) in Johns Hopkins (JH) Medicine (Baltimore, MD). Only gynecologic surgeons without obstetric duties were included. Physicians who were not faculty, such as clinical associates, and part-time faculty were excluded from the study.

### Data collection

We collected data from the EHR (Epic, Epic Systems, Verona, WI) using Signal, a provider efficiency tracking tool (Epic Systems) that provides data on ambulatory care. Signal enables data collection about how clinicians use the EHR using a standardized method across study subjects. Data were collected for January to December 2023.

We measured the surgeons’ clinical workload by their daily number of scheduled clinical appointments and hours to assess for size differences in the women’s and men’s patient schedules. We focused on the amount of time that surgeons spent working outside of regular hours because this typically indicates uncompensated work that can impede on personal and family life. We also examined the amount of time that surgeons spent doing different EHR tasks that are directly relevant to patient care, including conducting clinical review activities and responding to In Basket messages. The In Basket is Epic’s primary communication method for surgeons to send and receive messages from patients, physicians and staff, and the EHR system [11]. We evaluated the surgeons’ ability to use the EHR through a proficiency score calculated by Signal and by analyzing the length of clinical notes as an indicator of efficiency with documentation. Finally, we assessed the contribution that surgeons received for notes and orders from other members of the clinical team. Table 1 provides details about the study metrics. We obtained data about the surgeons’ self-identified genders, race, and ethnicity from the JH University School of Medicine’s Office of Faculty Information. The JH University Institutional Review Board approved this study, acknowledged all study procedures as exempt research, and waived the requirement for consent (protocol IRB00365761). This project was not registered as a clinical trial.

**Table 1:** Metric definitions

Metrics for clinical workload	Definition
Appointments per day	Average number of clinic appointments per day
Percentage of days with ≥ 1 appointment	Percentage of days with at least one clinic appointment
Scheduled clinical hours per day	Average clinical time scheduled per day
**Metrics for EHR work outside of regular hours**	**Definition**
Minutes outside of scheduled clinical hours	Average time spent working in the EHR outside of scheduled clinic hours. This includes a 30-min buffer before the start of the first scheduled appointment and after the last appointment concludes
Minutes outside of 7 AM to 7 PM	Average time spent working in the EHR on scheduled clinic days outside of 7 AM to 7 PM
Minutes on days without scheduled patients	Average time spent working in the EHR on days with no scheduled patients
**Metric for specific EHR tasks**	**Definition**
Minutes in notes	Average time spent writing notes per appointment
Minutes in In Basket messages	Average time spent reading and responding to In Basket messages per appointment
Minutes in orders	Average time spent writing orders per appointment
Minutes in clinical review	Average time spent doing clinical review activities per appointment
In Basket messages received per day	Average number of In Basket messages received per day
**Metrics for EHR proficiency**	**Definition**
Proficiency score	Determined by how often the surgeon used Epic efficiency tools: • 0.01 points per QuickAction used, up to 100 uses • 0.01 points per provider preference list entry, up to 100 entries • 0.2 points per 10% of notes written using SmartTools • 2 points for having customized level of service speed buttons • 2 points for having customized diagnosis speed buttons • 2 points for using Chart Search
Notes documentation length	Average number of characters per note
**Metrics for EHR support from clinical team members**	**Definition**
Percentage of notes written by a clinical team member	Text written by a clinical team member other than the faculty surgeon (excludes letters and patient instructions)
Percentage of orders started by a clinical team member	Orders signed by the faculty surgeon that were pended by a different clinical team member

Epic Systems. Metric Reference. https://signal.epic.com/Documentation/MetricReference#Metric-1152

### Statistical analysis

We examined gender differences in clinical and EHR workload using t-tests. Then we evaluated potential gender differences in clinical and EHR workload using mixed effects linear regressions adjusted for differences within specialty (with gender as a fixed effect and specialty as a random effect). Gender differences among the surgical specialties were analyzed by chi-square tests. We assumed significance at *p* < 0.05 and conducted the analyses using Stata version 13.0 (StataCorp, College Station, TX).

## Results

We evaluated EHR use from 323 surgeons (32% women). These included 2 women and 2 men instructors; 57 women and 72 men assistant professors; 29 women and 73 men associate professors; and 14 women and 74 men professors. The surgeons had been at rank for a mean of 6.4 years (standard deviation [SD]: 7.1). 88% were not Hispanic, 5% were Hispanic, and 7% preferred to not disclose their ethnicity. The cohort’s racial makeup was 59% White; 26% Asian; 7% Black or African-American; 1% American Indian, Alaska Native, Native Hawaiian, or Other Pacific Islander; and 7% did not disclose their race.

Table 2 shows surgeon gender by specialty. Urology, orthopedic, and neurosurgery had the largest proportions of men surgeons, and gynecologic surgery had the most women surgeons. Their clinical workload did not differ by surgeon gender in the unadjusted or adjusted analyses. This included the average number of clinical appointments per day, percentage of days with at least one scheduled clinical appointment, and the number of scheduled clinical hours per day (all comparisons *p* > 0.05; Table 3).

**Table 2:** Surgeon gender

Specialty, *n* (%)	Women	Men	*p*-value ^a^
Otolaryngology	11 (29)	27 (71)	< 0.001*
General surgery^b^	30 (33)	61 (67)	
Neurosurgery	4 (16)	21 (84)	
Gynecologic surgery^c^	15 (88)	2 (12)	
Ophthalmology	24 (37)	41 (63)	
Orthopedic surgery	8 (17)	39 (83)	
Plastic surgery	6 (33)	12 (67)	
Urology	4 (18)	18 (82)	
**Total**	102 (32)	221 (68)	

EHR, electronic health record. *p < 0.05. ^a^Chi-square test. ^b^Includes vascular, minimally invasive, pediatric, cardiac, pediatric cardiac colorectal, thoracic, hepatobiliary, acute care and trauma, transplant, surgical oncology, surgical specialties. ^c^Only gynecologic surgeons without obstetric duties were included

**Table 3:** Clinical and electronic health record workload

	Women	Men	*p*-value	
**Clinical workload, mean (SD)**			**Unadjusted** ^a^	**Adjusted** ^b^
Number of clinical appointments per day	13.3 (7.2)	14.6 (9)	0.206	0.087
Percentage of days with ≥ 1 clinical appointment	20 (10)	20 (10)	0.875	0.992
Scheduled clinical hours per day	4.3 (1.6)	4.5 (1.8)	0.207	0.198
**Minutes spent working in the EHR per day, mean (SD)**				
Outside of scheduled clinical hours	27.8 (17.3)	20 (14.1)	< 0.001*	< 0.001*
Outside of 7 AM to 7 PM	15.1 (12.5)	11.3 (10.2)	0.005*	0.007*
On days without scheduled patients	28.4 (15.9)	22.1 (14.3)	0.001*	0.016*
**Minutes spent on individual EHR tasks per appointment, mean (SD)**				
Notes	5.2 (4)	3.4 (2.7)	< 0.001*	< 0.001*
In Basket messages	1.9 (1.6)	1.2 (1.2)	< 0.001*	0.004*
Orders	1.1 (0.8)	0.9 (0.7)	0.009*	0.120
Clinical review	2.3 (1.8)	1.7 (1.4)	0.002*	0.032*
**Number of In Basket messages received per day and the message source, mean (SD)**				
Total	19.4 (10.5)	20.1 (13.3)	0.663	0.351
EHR system	8 (6.3)	8.5 (5.9)	0.555	0.925
Physicians and staff	6.8 (8.7)	6.6 (10.8)	0.851	0.325
Patients	2.4 (2)	2.3 (2.8)	0.866	0.870
**EHR proficiency, mean (SD)**				
Proficiency score	5 (1.6)	5.1 (1.6)	0.478	0.144
Notes documentation length (characters per note)	4127.9 (2516.2)	4050.5 (2575)	0.802	0.702
**Support from clinical team members, mean (SD)**				
Percentage of notes written by another clinical team member (% text)	10 (30)	20 (30)	0.015*	0.138
Percentage of orders started by another clinical team member (%)	30 (30)	30 (30)	0.275	0.244

EHR, electronic health record. SD, standard deviation. *p < 0.05. ^a^T-test. ^b^Mixed effects linear regressions adjusted for differences within specialty (with gender as a fixed effect and specialty as a random effect)

However, the amount of time spent working in the EHR outside of regular working hours significantly differed by gender after adjusting for specialty. Women surgeons spent more time than men working in the EHR outside of their scheduled clinical hours (women: mean 27.8 min [SD: 17.3], men: 20.0 [14.1], *p* < 0.001). Women also conducted more of their EHR tasks during hours outside of 7 AM to 7 PM (women: 15.1 min [12.5], men: 11.3 [10.2], *p* = 0.007) and on days without scheduled patients (women: 28.4 min [15.9], men: 22.1 [14.3], *p* = 0.016) relative to men.

The specific EHR tasks with gender differences varied. Women surgeons spent more time working on notes (women: 5.2 min per appointment [4], men: 3.4 [2.7], *p* < 0.001). The amount of time women spent conducting clinical review activities also exceeded that of men (women: 2.3 min per appointment [1.8] men: 1.7 [1.4], *p* = 0.032). However, time spent writing orders was not related to gender after adjusting for specialty (*p* > 0.05).

Work conducted for messaging through the EHR system contrasted by surgeon gender. Women spent more time working on In Basket messages (women: 1.9 min per appointment [1.6], men: 1.2 [1.2], *p* = 0.004) though the total number of received messages did not differ (*p* > 0.05). The number of messages delivered to the surgeons from patients, physicians or staff, or the EHR system also were not associated with surgeon gender (*p* > 0.05 for each).

Men and women had similar ability with documenting in the EHR. More specifically, the EHR proficiency scores and the number of characters documented per note were not related to gender in the unadjusted or adjusted analyses (*p* > 0.05 for all comparisons). Women and men also received similar assistance with their EHR work because the percentage of notes or orders written by clinical team members did not differ between women and men after adjusting the analysis for specialty (*p* > 0.05).

## Discussion

Time spent documenting in the EHR is typically uncompensated, does not meaningfully advance surgeons’ careers, and may contribute to burnout. As such, the scope of the EHR gender gap must be clarified. We evaluated the potential for surgeon gender differences in the EHR ambulatory workload from eight departments over one year at our academic medical institution. We found that women surgeons spent more time than men working in the EHR outside of regular hours. This difference occurred despite no difference in clinical workload by gender. We did not observe any differences in support for EHR work by other clinical team members, number of patient messages, documentation length, or surgeon proficiency with the EHR. The greater EHR work for women appeared to be due to time spent on notes, messages, and clinical review. Thus, our hypothesis that gender differences in EHR use would be associated with EHR support, patient message volume, and note length was not supported. To the authors’ knowledge, our study is the largest to date about gender disparities and the EHR among surgeons and surgical subspecialists. Results from the current and published [7, 8] studies will inform the design of a potential multi-center trial about surgeon gender differences in EHR work.

The differences between women’s and men’s EHR use carries a cumulative impact that may substantially reduce women’s academic productivity and impair their well-being. For instance, assuming 16 clinical days/month, 8 min/day of additional EHR work outside of scheduled clinical hours for women translates to approximately 26 h/year of lost time relative to men. This time could instead be spent on research, teaching, disseminating scholarly work, and supporting personal and family well-being. Women physicians are disproportionately affected by burnout compared to men physicians [12]. Burnout decreases professional satisfaction and quality of life [13], and it may raise health system costs [14]. It is also detrimental to patient care and safety [15]. Moreover, extra EHR work for women may contribute to gender disparities in surgical leadership [9], academic productivity [16], promotion [16], and salary [10] by making it difficult for women to find time to engage in leadership development and do promotable work.

Our findings generally agree with two other published studies [7, 8] that reported greater EHR work for women surgeons outside of regular work hours than men, though these studies had fewer surgeons and excluded gynecologic and ophthalmologic surgeons. We included these specialties to comprehensively evaluate EHR use by all surgeons at our institution. It is worth noting differences among the studies. At our institution, women and men surgeons had a similar clinical appointment workload with no difference in note length. By contrast, Malacon et al. [8]. found that men surgeons had more clinical appointments and wrote shorter notes than women. These institutional differences emphasize the importance of studying surgical EHR differences at multiple centers to comprehensively evaluate the problem without inadvertently implying globally that women surgeons carry a lighter clinical workload than men [8]. 

The cumulative surgical data from our and published studies [7, 8] are similar to that from nonsurgical medical specialties showing women bear a greater EHR workload outside of regular work hours than men [16–21]. Thus, gender inequity with the EHR appears to be a widespread problem that should be reviewed and addressed at the national level. A common data collection method across institutions would allow the EHR gender gap to be tracked over time. We used Signal in our study because it allows for the standardization of Epic data collection. More than 60% of EHRs in the United States are from Epic [17]. 

We specifically studied gender differences in clinical support for EHR work, note length, and EHR proficiency because these are modifiable factors. However, none were related to surgeon gender. Thus, women conduct their EHR work outside of regular working hours due to other issues. One contributing factor could be an unequal distribution of household responsibilities; women surgeons often carry more family and household duties than men surgeons [18]. How family and household obligations influence when surgeons can do their EHR work deserves study. Published research indicates that many women surgeons and physicians still do their family and household tasks– including family care, meal preparation, cleaning and other household duties– in addition to their professional work [19–21]. Thus, many women surgeons must do their EHR documentation and answer patient messages outside of regular working hours, including very early in the morning and late at night.

Methods to support surgeons who have family care responsibilities [22] include providing mentorship, coaching, and open discussions about work-life integration. Institutional assistance with finding high-quality child or elderly care with extended hours and pro-rating clinical requirements after childbirth recovery and parental leave would support surgeons of all genders. Supervisors must ensure equitable distribution of clinical duties like call and weekend shifts. Time banking systems [23] can provide credits to surgeons who do uncompensated work, such as filling in for clinical shifts, serving on committees, and mentoring trainees. These credits could be redeemed for support services that range from housecleaning and errand outsourcing to grant and manuscript editing.

Department chairs must be aware of the EHR gender gap and partner with institution and division leadership to mitigate the problem. Women and men should be assigned equal responsibility for uncompensated work, such as coordinating clinical and lecture schedules. The distribution of qualified and motivated administrators and clinicians to support surgeons must also be equitable. Because men surgeons often have higher academic rank than women [16], rank should not influence the distribution of skilled administrative and clinical support.

Men surgeons hold more leadership positions than women [9, 24], and this might influence the relationship between gender and EHR work. For instance, leaders often have high administrative responsibilities during the day, thereby making surgeon leaders do their EHR work outside of regular hours. But holding a leadership position may decrease a surgeon’s assigned clinical workload. We could not evaluate the distribution of leadership roles by gender because comprehensive data of all leadership positions in our eight surgical departments are not centrally available in the JH School of Medicine. Nonetheless, we found that women surgeons had a greater EHR documentation burden outside of regular work hours relative to men independent of clinical workload.

We also observed that women surgeons spent significantly more time than men working on notes, clinical review, and messages to patients and other clinicians. Evaluating the quality of patient care was beyond the scope of our study. The relationship between patient outcome and surgeon gender is unclear. Some studies indicate that women surgeons have better patient outcomes [25], particularly for elective surgeries [26], whereas other studies show no effect of surgeon gender on patient satisfaction [27]. Patients may also expect more communication with women surgeons relative to men surgeons [5]. Future research should evaluate whether time spent on different EHR tasks is related to clinical outcomes. The future impact of generative artificial intelligence [28] on the efficiency of documentation, answering messages, and clinical review in the EHR should also be assessed.

Importantly, education about the existence of the EHR gender gap should be discussed at surgical society meetings and among institutional leaders to invite collective solutions. The data must be measured over time to ensure that the gap decreases. Tracking EHR data with the same vigilance that the American Association of Medical Colleges reports gender differences in salary, rank, and high-level leadership [29–31] might encourage institutions to reduce the EHR gender gap or provide compensation when afterhours EHR work is necessary.

Our study focused on surgical ambulatory work to ensure consistency in the methodology. However, this comprises only a portion of most academic surgeons’ jobs. Research on potential gender differences in the EHR workload for inpatient surgical care, including dictation, is needed to propose methods that mitigate burnout [12, 32] and delays in professional advancement [9, 16, 24], which women currently experience to a greater degree than men.

We acknowledge several study limitations, including the retrospective single-center design. We limited the study period to avoid confounders from operational changes at our institution after the COVID-19 pandemic. Our data also do not include percentages of clinical effort or EHR work conducted using mobile apps, which should be incorporated into future research. Future multi-center studies should examine the relationships between surgeon gender and EHR work when surgeons are on inpatient service and covering emergencies. An additional important area for future research is the relationship between gender, EHR workload, and revenue value unit generation.

## Conclusions

Women surgeons had a greater EHR burden outside of regular hours than men despite similar clinical workload, support for EHR work from clinical team members, number of patient messages, note length, and EHR proficiency. The additional EHR work for women was partly attributed to spending more time doing clinical reviews, writing notes, and answering messages from patients, other clinicians, and staff. These data will contribute to designing a potential multi-center trial to evaluate the prevalence of the surgical EHR gender gap at institutions across the United States.

## Data Availability

The datasets used and analyzed for this study are available from the corresponding author on reasonable request.
